# Supplementary material for: Harvesting Mango Fruit with a Short Stem-End Altered Endophytic Microbiome and Reduce Stem-End Rot
Source: Microorganisms. 2020 Apr 13;8(4):558. doi: 10.3390/microorganisms8040558 (PMC7232454; doi:10.3390/microorganisms8040558)
Supplement: Supplementary file 1 [file microorganisms-08-00558-s001.pdf]

Harvesting mango fruit with a short stem-end altered endophytic microbiome and reduce stem-end rot

Ortal Galsurker[1], Sonia Diskin [1], Danielle Duanis-Assaf[1], Adi Doron-Faigenboim[2], Dalia Maurer[1], Oleg Feygenberg[1], and Noam Alkan[1,*]

Supporting materials:

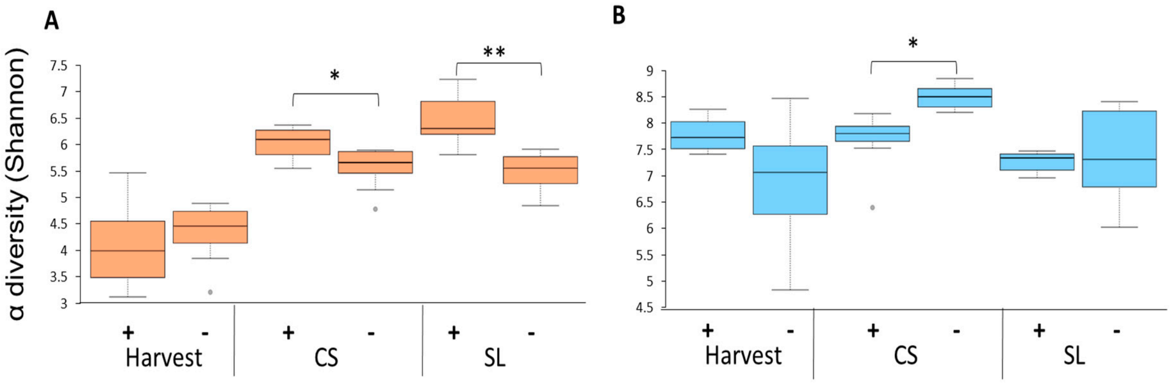

Figure S1. Statistical analysis of microbial α-diversity that conducted by Shannon Index. A. Fungal diversity. B. Bacterial diversity. Asterisks indicate significant differences in Shannon diversity that estimates between harvest technique in each time point.

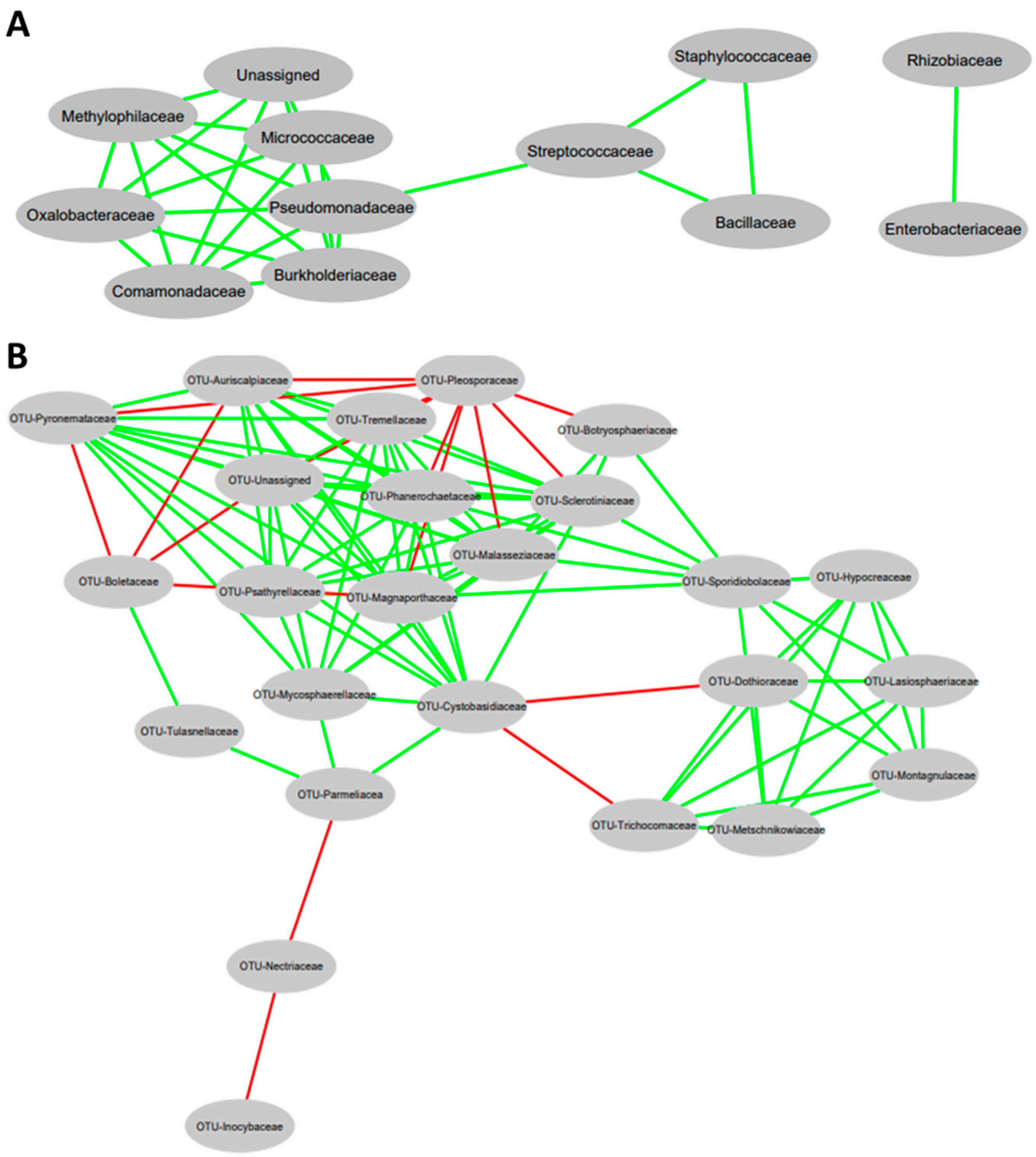

Figure S2. Co-occurrence network structures of the relationships among endophytic bacterial (A) and fungal (B) families in mango stem-end. The green and red lines indicate significant positive and negative correlations between two families, respectively.

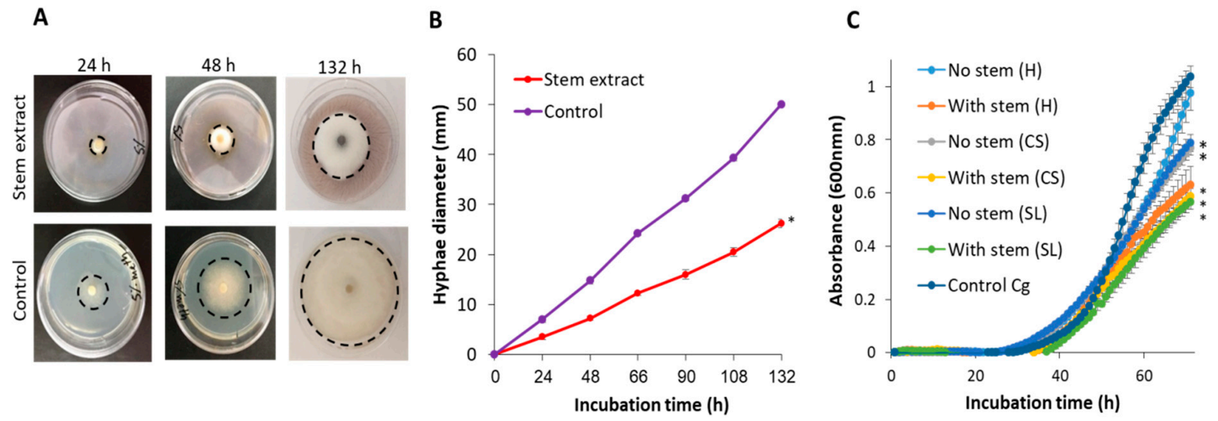

Figure S3. Effect of mango stem-end extract on *C. gloeosporioides* growth. Stem-end of 'Shelly' mango fruit was organically extracted for evaluating the antifungal activity against *C. gloeosporioides*. A and B. Representative pictures and *in-vitro* growth curves of *C. gloeosporioides* on PDA solid media embedded with stem extract over 132h. B. *In vitro* growth rate of *C. gloeosporioides* conidia in the presence of different extracts (from fruit harvested with and without stems) at harvest (H), after cold storage (CS) and after shelf life (SL). The rate of fungal growth in the different extract was compared to control. Each sample was averaged and background-corrected. Data presented as mean ± SE (n = 6). Asterisk (*) represents a statistically significant difference between the different extracts and control, using t-test, $p \leq 0.001$.

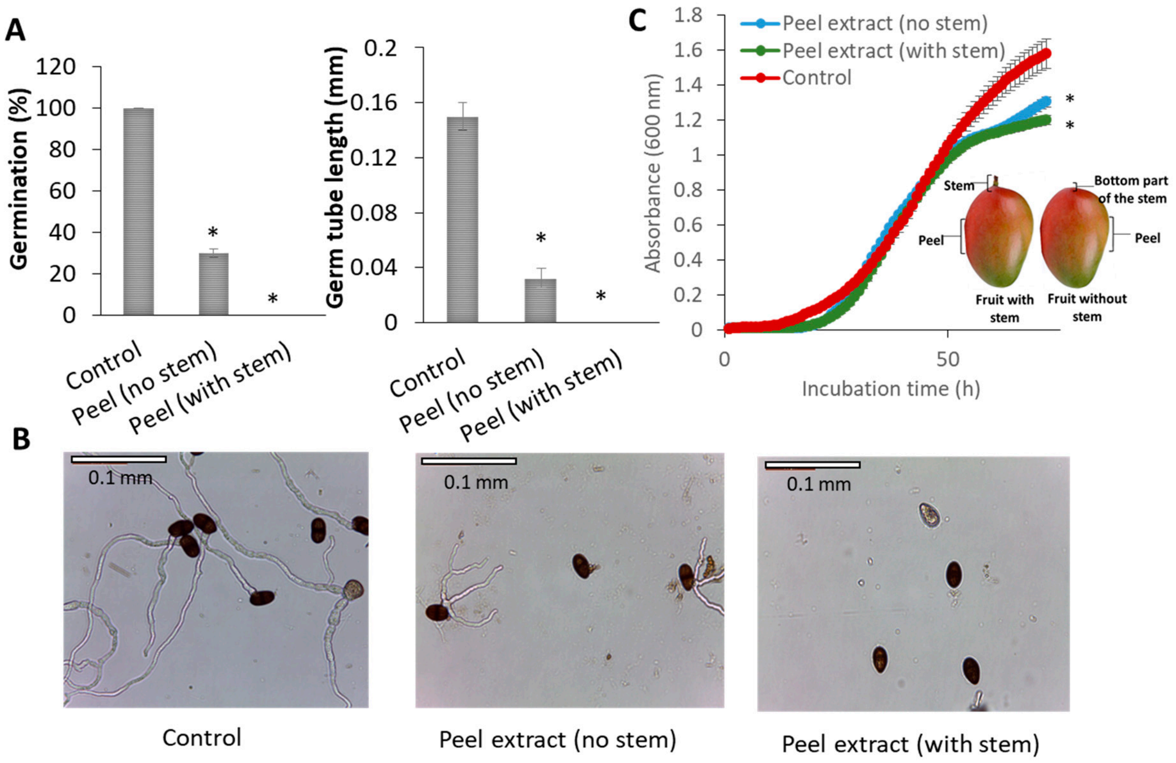

Figure S4. Effect of mango peel extracts from fruits with and without stem on *L. theobromae* germination and growth. Peels of 'Shelly' mango fruits were organically extracted for evaluating the antifungal activity against the fungal pathogen *L. theobromae*. The effect of different extracts (in 1% methanol) on conidia germination was evaluated by microscopic determination after 20 h of incubation. A. Percentage germination (left section) and germ tube length (right section) of *L. theobromae*. B. Microscopic images (40X magnification) showing *L. theobromae* germination inhibition by peel extract, compared to the control. C. *In vitro* growth rate of *L. theobromae* in microplates in the presence of the different extracts. Each sample was averaged and background-corrected. Data presented as mean ± SE (n = 6). Asterisk (*) represents a statistically significant difference between the different extracts and control, using t-test, p≤0.001. H; harvest, CS; cold storage and SL; shelf life.

Table S1. Evaluation of fruit quality parameters and decay incidences in “Noa” and “Kent” mango cultivars harvested with or without stem.

	Storage time	Noa Mango (2019)		Kent Mango (2019)	
		No Stem	With Stem	No Stem	With Stem
**Firmness (index 1-10)**	CS	9.2 ±0.1	9.8 ±0.1	9.8 ±0.1	10.0 ±0.0
	SL	3.1 ±0.2	3.5 ±0.1	1.3 ±0.1	2.2 ±0.2
**Yellowing (index 1-10)**	CS	3.1 ±0.2	2.0 ±0.1	1.6 ±0.2	1.1 ±0.1
	SL	9.7 ±0.1	9.9 ±0.1	6.8 ±0.1	6.2 ±0.2
**Color (hue)**	H	125.2 ±0.8	125.2 ±0.8	124.9 ±1.8	124.9 ±1.8
	CS	116.6 ±2.1	122.2 ±1.8	121.8 ±3.1	122.6 ±5.6
	SL	83.4 ±3.1	80.9 ±1.5	111.8 ±5.4	112.7 ±4.4
**TSS (% brix)**	H	8.2 ±0.3	8.2 ±0.3	7.6 ±0.2	7.6 ±0.2
	CS	10.5 ±0.4	12.6 ±0.7	19.1 ±0.4	17.5 ±0.1
	SL	14.1 ±0.6	14.9 ±0.2	18.1 ±0.3	18.1 ±0.4
**Acid (%)**	H	1.0 ±0.1	1.0 ±0.1	0.9 ±0.1	0.9 ±0.1
	CS	0.9 ±0.1	0.9 ±0.1	0.8 ±0.1	0.8 ±0.1
	SL	0.4 ±0.0	0.4 ±0.1	0.5 ±0.1	0.6 ±0.0
**SER (%)**	CS	0±0	0±0	2.5±2.5	0±0
	SL	21.9±2.7	0±1	9.6±4.2	2.2±2.2
**Side rot (%)**	CS	2.5±2.5	0±0	0±0	0±0
	SL	8.2±5.6	0±1	16.8±4.4	11.7±5.3

The fruits were evaluated after three weeks in cold storage at 12°C (CS) followed by shelf life (SL) storage at 20°C. Fruit quality parameters: Fruit Brix (%TSS), Acid (% citric acid equivalence), color (hue), yellowing (index 1-10; 1-green, 10-yellow), firmness (index 1-10), and the incidence of SER and side rot (%).

Table S2. Sequence statistics of ITS libraries after processing.

Sample	Trimmed on quality		Reads missing primers		Ambiguity discards		Short length discards		Chimeric sequences		Usable reads	
**H - no stem (a)**	1203	2.14%	3023	5.37%	14	0.02%	8977	15.96%	28	0.06%	44202	78.58%
**H - no stem (b)**	824	1.73%	1597	3.35%	10	0.02%	3630	7.63%	248	0.90%	27172	88.10%
**H - no stem (c)**	744	1.86%	1363	3.41%	9	0.02%	5143	12.87%	359	1.41%	25073	82.29%
**H - no stem (d)**	713	2.44%	1032	3.54%	6	0.02%	4329	14.84%	76	0.59%	12704	81.01%
**H - no stem (e)**	661	1.03%	1726	2.70%	23	0.04%	1090	1.71%	255	1.25%	20074	94.31%
**H - no stem (f)**	1051	2.03%	1881	3.63%	15	0.03%	3204	6.18%	432	1.31%	32574	88.85%
**H - no stem (g)**	2332	1.85%	3464	2.75%	28	0.02%	20085	15.93%	128	0.68%	18792	80.62%
**H - no stem (h)**	1436	2.26%	2348	3.70%	12	0.02%	4054	6.39%	58	0.23%	25081	89.66%
**H - with stem (a)**	239	2.70%	425	4.80%	3	0.03%	2390	27%	6	0.10%	6029	68.07%
**H - with stem (b)**	1141	1.76%	2098	3.24%	22	0.03%	4130	6.39%	50	0.08%	61037	90.25%
**H - with stem (c)**	843	1.06%	1831	2.30%	22	0.03%	3333	4.19%	102	0.18%	56914	93.29%
**H - with stem (d)**	1012	0.80%	2723	2.16%	25	0.02%	1010	0.80%	19	0.19%	9955	96.82%
**H - with stem (e)**	815	1.33%	1679	2.74%	19	0.03%	1780	2.90%	183	0.26%	70479	94.07%
**H - with stem (f)**	1184	1.27%	3290	3.53%	30	0.03%	4806	5.15%	48	0.06%	74240	91.23%
**H - with stem (g)**	1990	1.88%	3023	2.85%	24	0.02%	15179	14.33%	91	0.73%	12306	82.06%
**H - with stem (h)**	2089	2.62%	2987	3.75%	23	0.03%	13800	17.34%	148	0.29%	51288	78.59%
**CS - no stem (a)**	810	2.46%	1281	3.89%	10	0.03%	3857	11.72%	127	0.24%	52746	84.11%
**CS - no stem (b)**	559	1.57%	1108	3.10%	10	0.03%	1593	4.46%	242	1.07%	22465	91.34%
**CS - no stem (c)**	430	1.84%	798	3.42%	5	0.02%	2184	9.37%	75	0.07%	102460	87.12%
**CS - no stem (d)**	461	1.69%	901	3.30%	5	0.02%	1577	5.77%	183	0.74%	24642	90.17%
**CS - no stem (e)**	774	3.20%	1124	4.64%	4	0.02%	4155	17.17%	8	0.08%	9737	78.09%
**CS - no stem (f)**	628	2.15%	996	3.41%	6	0.02%	2768	9.48%	73	0.27%	26881	86.82%
**CS - no stem (g)**	1996	2.54%	2967	3.77%	16	0.02%	7899	10.03%	368	0.32%	114267	85.86%
**CS - no stem (h)**	8	10.67%	48	64%	0	0%	3	4%	92	0.11%	85077	31.89%
**CS - with stem (a)**	1306	3.53%	1631	4.41%	8	0.02%	7938	21.46%	59	0.09%	62721	74.02%
**CS - with stem (b)**	798	2.27%	1229	3.49%	8	0.02%	3970	11.27%	109	1%	10815	84.21%
**CS - with stem (c)**	512	3.40%	611	4.06%	6	0.04%	3523	23.39%	28	0.12%	23775	72.40%
**CS - with stem (d)**	540	2.67%	774	3.83%	3	0.01%	3436	17.01%	11	0.12%	9305	79.03%
**CS - with stem (e)**	744	4.25%	694	3.96%	2	0.01%	4423	25.25%	0	0%	24	70.78%
**CS - with stem (f)**	491	3.25%	530	3.51%	2	0.01%	3351	22.19%	38	0.27%	14188	74.01%
**CS - with stem (g)**	500	3.10%	606	3.75%	9	0.06%	2750	17.03%	91	0.19%	46648	78.97%
**CS - with stem (h)**	1137	3.87%	1222	4.16%	7	0.02%	5418	18.46%	229	0.83%	27523	76.53%
**SL - no stem (a)**	994	2.18%	1616	3.55%	5	0.01%	4763	10.46%	80	0.40%	20127	85.58%
**SL - no stem (b)**	680	2.39%	1181	4.16%	6	0.02%	4931	17.35%	79	0.14%	58336	78.33%
**SL - no stem (c)**	602	1.87%	1215	3.78%	6	0.02%	3969	12.35%	120	0.10%	121925	83.75%
**SL - no stem (d)**	500	0.93%	1360	2.53%	5	0.01%	937	1.74%	41	0.35%	11762	95.37%
**SL - no stem (e)**	502	0.91%	1461	2.64%	4	0.01%	1041	1.88%	56	0.25%	22244	95.22%
**SL - no stem (f)**	562	1.99%	1082	3.83%	3	0.01%	2028	7.18%	577	0.85%	67261	88.13%
**SL - no stem (g)**	2751	2.16%	4638	3.65%	21	0.02%	7783	6.12%	115	0.20%	57763	90.01%
**SL - no stem (h)**	2072	2.61%	2823	3.56%	9	0.01%	5842	7.36%	58	0.07%	87641	89.00%
**SL - with stem (a)**	952	4.28%	998	4.49%	2	0.01%	6996	31.48%	80	0.19%	42284	63.83%
**SL - with stem (b)**	649	2.14%	1032	3.40%	12	0.04%	2400	7.90%	109	0.97%	11108	87.69%
**SL - with stem (c)**	701	2.95%	1104	4.64%	5	0.02%	2980	12.52%	129	0.33%	39025	82.49%
**SL - with stem (d)**	643	4.74%	754	5.55%	3	0.02%	2844	20.95%	24	0.07%	33420	73.40%
**SL - with stem (e)**	671	4.05%	878	5.31%	2	0.01%	3867	23.37%	179	1.12%	15813	70.20%
**SL - with stem (f)**	821	3.36%	1326	5.43%	5	0.02%	2864	11.74%	77	0.39%	19636	82.42%
**SL - with stem (g)**	494	3.73%	1070	8.09%	2	0.02%	2842	21.48%	246	0.82%	29760	69.60%
**SL - with stem (h)**	486	3.51%	784	5.66%	1	0.01%	3324	23.99%	76	0.28%	26854	70.06%
	44051		74332		477		215196		6010		1846153	

The reads of each sample were quality filtered as follow: (1) Ambiguous nucleotides (N) were trimmed from the ends, and reads with ambiguous internal nucleotides were discarded. (2) Primer sequences were trimmed from the reads. (3) Reads were cut using a quality threshold of $p = 0.01$. (4) Reads, after trimming, that were less than 100bp in length were discarded. (5) Chimeric sequences were filtered resulting in usable read.

Table S3. Sequence statistics of 16S libraries after processing.

Sample	Trimmed on quality		Reads missing primers		Ambiguity discards		Short length discards		Chimeric sequences		Usable reads	
H - no stem (a)	5735	9.65%	2097	3.53%	28	0.05%	6225	10.48%	13365	26.18%	37689	59.76%
H - no stem (b)	4745	9.88%	1684	3.51%	21	0.04%	5613	11.69%	45697	23.03%	152703	61.73%
H - no stem (c)	5209	10.50%	1992	4.02%	25	0.05%	6844	13.80%	35139	23.18%	116472	58.96%
H - no stem (d)	3105	9.88%	1153	3.67%	24	0.08%	3896	12.40%	30769	22.04%	108831	61.82%
H - no stem (e)	7353	10.49%	2812	4.01%	54	0.08%	7879	11.24%	7922	23.69%	25516	60.99%
H - no stem (f)	3865	9.80%	1632	4.14%	26	0.07%	4360	11.05%	24838	19.86%	100230	64.89%
H - no stem (g)	8282	9.37%	2277	2.58%	57	0.06%	6106	6.91%	46066	22.70%	156861	67.75%
H - no stem (h)	19695	8.95%	6392	2.90%	137	0.06%	15176	6.89%	60396	35.15%	111429	54.99%
H - with stem (a)	8735	11.85%	2604	3.53%	46	0.06%	8338	11.31%	11216	23.79%	35922	61.30%
H - with stem (b)	7082	12.49%	2136	3.77%	44	0.08%	6416	11.32%	15554	34.43%	29622	50.41%
H - with stem (c)	3812	11.20%	1631	4.79%	23	0.07%	3131	9.20%	8091	10.13%	71811	75.82%
H - with stem (d)	5434	10.08%	1848	3.43%	23	0.04%	4921	9.12%	64353	24.48%	198480	62.93%
H - with stem (e)	6254	11.75%	1878	3.53%	40	0.08%	6115	11.49%	4793	16.38%	24467	68.52%
H - with stem (f)	6417	11.25%	2309	4.05%	25	0.04%	5733	10.05%	54368	29.91%	127394	55.94%
H - with stem (g)	22448	11.49%	4951	2.54%	117	0.06%	18907	9.68%	17782	21.60%	64544	66.12%
H - with stem (h)	23067	11.74%	5145	2.62%	110	0.06%	19307	9.83%	13859	28.81%	34244	58.69%
CS - no stem (a)	8170	9.34%	2407	2.75%	39	0.04%	7045	8.05%	32172	22.90%	108310	66.25%
CS - no stem (b)	11530	10.17%	3302	2.91%	63	0.06%	9905	8.74%	59544	32.08%	126049	56.22%
CS - no stem (c)	18942	9.81%	5516	2.86%	114	0.06%	15663	8.11%	67233	28.65%	167424	60.32%
CS - no stem (d)	16223	8.82%	5539	3.01%	115	0.06%	13712	7.46%	57167	37.33%	95990	52.14%
CS - no stem (f)	17782	9.14%	5052	2.60%	106	0.05%	15181	7.80%	50347	22.95%	169007	66.60%
CS - no stem (g)	19450	8.76%	6384	2.87%	138	0.06%	17192	7.74%	71942	23.92%	228776	65.40%
CS - no stem (h)	16464	8.95%	4844	2.63%	120	0.07%	13879	7.54%	50123	29.42%	120242	60.34%
CS - with stem (a)	16725	11.65%	4088	2.85%	91	0.06%	14368	10%	63285	25.38%	186046	61.71%
CS - with stem (b)	27789	11.09%	7392	2.95%	156	0.06%	23586	9.42%	53564	30.73%	120744	56.84%
CS - with stem (c)	23424	8.95%	6981	2.67%	172	0.07%	18953	7.24%	22100	20.09%	87911	69.94%
CS - with stem (d)	29012	8.72%	8454	2.54%	226	0.07%	23481	7.05%	15605	20.05%	62216	70.29%
CS - with stem (e)	30803	10.37%	9013	3.03%	196	0.07%	25110	8.45%	23306	26.42%	64900	62.03%
CS - with stem (e)	17484	8.67%	5407	2.68%	138	0.07%	14248	7.07%	55528	23.56%	180176	66.62%
CS - with stem (f)	23816	8.61%	7817	2.82%	188	0.07%	19425	7.02%	51053	31.02%	113511	59.07%
CS - with stem (g)	24313	10.56%	6629	2.88%	135	0.06%	20540	8.92%	49794	30.15%	115346	57.99%
CS - with stem (h)	18850	10.92%	5154	2.98%	106	0.06%	15800	9.15%	17109	19.44%	70892	68.36%
SL - no stem (a)	9813	9.89%	2826	2.85%	60	0.06%	8142	8.20%	48081	32.28%	100863	56.61%
SL - no stem (b)	14443	9.89%	3886	2.66%	79	0.05%	11894	8.14%	15232	25.65%	44151	63.49%
SL - no stem (c)	20726	9.95%	5321	2.55%	129	0.06%	17220	8.27%	19687	23.88%	62766	65.23%
SL - no stem (d)	26167	9.94%	6934	2.63%	178	0.07%	21486	8.16%	12263	25.05%	36688	64.09%
SL - no stem (e)	15877	9.16%	4785	2.76%	84	0.05%	15331	8.84%	20353	21.77%	73152	66.58%
SL - no stem (f)	15372	9.23%	4701	2.82%	117	0.07%	12735	7.65%	42281	25.74%	121984	63.72%
SL - no stem (g)	19031	10.28%	5231	2.83%	110	0.06%	15557	8.40%	52622	30.71%	118705	58.00%
SL - no stem (h)	18431	9.58%	5277	2.74%	125	0.06%	16607	8.63%	4047	15.36%	22308	73.20%
SL - with stem (a)	9098	9.86%	2748	2.98%	53	0.06%	7061	7.65%	25265	32.40%	52717	56.92%
SL - with stem (b)	9212	9.98%	2662	2.88%	54	0.06%	7259	7.86%	31547	24.23%	98674	64.96%
SL - with stem (c)	11987	9.76%	3468	2.82%	69	0.06%	9263	7.54%	11529	28.32%	29175	61.26%
SL - with stem (d)	9954	9.53%	3231	3.09%	65	0.06%	7669	7.34%	58655	29.57%	139730	59.93%
SL - with stem (e)	16060	10.22%	4388	2.79%	108	0.07%	12170	7.74%	48245	28.07%	123646	61.32%
SL - with stem (f)	14729	9.48%	4300	2.77%	96	0.06%	11319	7.29%	21617	34.47%	41102	55.41%
SL - with stem (g)	8243	9.52%	2415	2.79%	65	0.08%	6246	7.22%	12176	29.88%	28573	60.04%
SL - with stem (h)	9590	9.78%	2666	2.72%	60	0.06%	7288	7.44%	34206	34.17%	65899	55.61%
	690748		201359		4355		584302		1651886		4573888	

The reads of each sample were quality filtered as follow: (1) Ambiguous nucleotides (N) were trimmed from the ends, and reads with ambiguous internal nucleotides were discarded. (2) Primer sequences were trimmed from the reads. (3) Reads were cut using a quality threshold of $p = 0.01$. (4) Reads, after trimming, that were less than 300bp in length were discarded. (5) Chimeric sequences were filtered resulting in usable reads.

Table S4. Significant changes in fungal and bacterial families across the conditions.

	Fungi (ITS)		Bacteria (16S)	
	**Family**	***p* value**	**Family**	***p* value**
**H/CS (with stem)**	Tremellaceae	2.22E-15	Sphingomonadaceae	4.40E-02
	Dothioraceae	2.47E-15	Methylophilaceae	9.89E-03
	Pleosporaceae	2.79E-14	Burkholderiaceae	1.80E-04
**H/SL (with stem)**	Tremellaceae	3.11E-15	Sphingomonadaceae	2.19E-03
	Dothioraceae	1.73E-06	Methylophilaceae	4.60E-03
	Metschnikowiaceae	1.23E-04	Micrococcaceae	1.24E-02
	Pleosporaceae	8.37E-03	Prevotellaceae	1.13E-02
**CS/SL (with stem)**	Pleosporaceae	1.65E-04	Micrococcaceae	2.29E-02
	Metschnikowiaceae	4.90E-04	Methylophilaceae	8.13E-03
	Botryosphaeriaceae	6.03E-03	Comamonadaceae	4.89E-02
	Nectriaceae	1.41E-02	Burkholderiaceae	1.67E-04
**H/CS (no stem)**	Tremellaceae	6.04E-08	Sphingomonadaceae	5.88E-04
	Pleosporaceae	5.07E-06	Methylophilaceae	9.67E-04
	Dothioraceae	3.64E-04	Bacillaceae	1.86E-02
			Comamonadaceae	7.78E-03
**H/SL (no stem)**	Tremellaceae	7.51E-14	Rhizobiaceae	4.20E-02
	Dothioraceae	4.28E-08	Sphingomonadaceae	5.00E-02
	Metschnikowiaceae	2.02E-06		
	Pleosporaceae	1.24E-03		
	Trichocomaceae	2.20E-04		
	Botryosphaeriaceae	3.80E-02		
**CS/SL (no stem)**	Metschnikowiaceae	8.09E-07	Methylophilaceae	2.10E-04
	Pleosporaceae	4.98E-06	Sphingomonadaceae	1.19E-02
	Dothioraceae	5.05E-04	Bacillaceae	1.94E-02
			Rhizobiaceae	1.49E-02
**With stem/no stem (H)**			Sphingomonadaceae	1.60E-02
			Micrococcaceae	1.27E-02
**With stem/no stem (CS)**			Bacillaceae	1.87E-02
			Micrococcaceae	2.38E-02
**With stem/no stem (SL)**	Metschnikowiaceae	1.04E-05	Sphingomonadaceae	1.17E-02
	Trichocomaceae	4.06E-04	Rhizobiaceae	2.34E-02
	Dothioraceae	2.29E-03		

$P \leq 0.05$ were considered as signifiacnt change.
